# The Association between Community Food Environments and Health Outcomes in High-Income Countries: A Systematic Literature Review

**DOI:** 10.1007/s13668-025-00662-z

**Published:** 2025-05-31

**Authors:** Alemayehu Digssie Gebremariam, Katherine Kent, Karen Charlton

**Affiliations:** 1 School of Medical, Indigenous and Health Sciences, Faculty of Science, Medicine and Health, University of Wollongong, Wollongong, NSW Australia; 2 Department of Public Health, College of Health Sciences, Debre Tabor University, Debre Tabor, Ethiopia

**Keywords:** Community food environment, High-Income Countries, Diabetes, Cardiovascular Diseases, Mortality, Systematic Review

## Abstract

**Purpose of the Review:**

Unhealthy diets are a major modifiable factor contributing to chronic noncommunicable diseases and are highly influenced by the surrounding community food environment. This review aimed to summarize and synthesize the existing published evidence on the relationship between the community food environment and health outcomes in high-income countries.

**Recent Findings:**

A systematic literature review using five databases was conducted and reported according to PRISMA guidelines (Registration number: CRD42023463886). Data were extracted from 55 studies and summarised using narrative synthesis due to heterogeneity. A harvest plot was used to depict the associations between the community food environment and health outcomes for various “healthy” and “unhealthy” food environment metrics. Among 55 included studies, the most researched health outcomes were diabetes (n = 31; 56.4%), cardiovascular diseases (n = 22; 40%) and chronic disease-associated mortality (n = 8; 14.6%). The density of fast-food outlets was predominantly positively associated with diabetes (n = 14/24 associations), cardiovascular diseases (n = 14/27), and chronic disease-associated mortality (n = 5/6). Similarly, the presence of fast-food outlets (n = 7/9), the density of convenience stores (n = 7/13), and the ratio/proportion of unhealthy food outlets (n = 4/4) were predominantly positively associated with diabetes. Conversely, the density of full-service restaurants (n = 8/12) was predominantly negatively associated with diabetes.

**Summary:**

Emerging evidence suggests an association between the community food environment and health outcomes, including diabetes, cardiovascular diseases, and chronic disease-associated mortality. A lack of consistency in metrics used to characterise the community food environment remains a significant challenge to informing evidence-based policies and programs to improve community health outcomes.

**Supplementary Information:**

The online version contains supplementary material available at 10.1007/s13668-025-00662-z.

## Introduction

Chronic noncommunicable diseases and their associated mortality remain the most significant public health concerns in high-income countries [1]. For example, populations in high-income countries continue to have the highest number of cardiovascular risk factors when compared with middle and low-income countries [2]. Further, in 2019, the prevalence of diabetes in adults was 10.4% and is projected to increase to 11.9% by 2045 in high-income countries [3].

A modifiable factor contributing to chronic noncommunicable disease risk is poor diet, which is highly influenced by the surrounding community food environment [4]. Community food environments are defined by an individuals'proximity to various types of food outlets or the density and diversity of different categories of food outlets within a designated geographical region [5]. These food environments encompass a wide range of considerations, such as physical access to food, economic considerations that impact financial access to food, policy frameworks that influence food availability and acceptability, and socio-cultural influences such as social norms and media influence [6]. The culmination of all these factors play a pivotal role in shaping food consumption behaviours [6].

The increasingly globalised food systems in high-income countries have resulted in considerable changes to community food environments, with far-reaching implications for population nutrition and food security [7]. Through the proliferation of multinational food corporations, such as major supermarkets and fast-food chains, globalised food systems have provided consumers with easier access to range of ultra-processed and ready-to-use foods that tend to be energy dense and nutrient poor [8, 9]. For instance, in Australia, Coles and Woolworths (two major supermarket chains) held 63% of the retail market in 2023 [10]. While supermarkets and grocery stores offer a large selection of fresh and healthy foods, they are also a primary source of many unhealthy foods [11].

The transition towards globalised food system in high-income countries has been associated with a decline in the overall healthiness of community food environments [12]. It is estimated that over 50% of the population in the USA and UK relies on ultra-processed and ready-to-use foods as their primary source of dietary energy [13, 14]. Global drivers that have shaped the contemporary food system have had a profound effect on chronic noncommunicable diseases and their risk factors, including obesity, diabetes and cardiovascular disease [15].

To date, there are a number of published systematic reviews providing evidence on an association between the food environment and obesity or dietary behaviour, which serve as proxy indicators of overall health outcomes [4, 16–19]. The most recent systematic review and meta-analysis on the association between food environment and obesity showed that the proximity of fast-food outlets was positively associated with obesity, whereas the density of fresh fruit and vegetable outlets and proximity to supermarkets inversely associated [20]. However, these reviews have largely overlooked direct health outcomes, such as metabolic and cardiovascular health, leaving a critical gap in understanding the broader impacts of the food environment on population health. Although two reviews have assessed the relationship between the community food environment and health outcomes, they were narrow in scope and their findings were inconclusive [21, 22]. For example, a systematic reviews on the association between the community food environment and diet, weight status or health in Australia, which included a limited number of studies focused on health outcomes, showed mostly null associations [21]. In addition, a systematic review of longitudinal studies on retail food outlet exposure and risk of metabolic syndrome, which focused exclusively on cohort studies and specific health outcome metrics, found limited evidence [22]. This demonstrates a need for more comprehensive reviews that directly examine the impact of the community food environment on specific health outcomes.

Therefore, the aim of this review was to summarise and synthesise the existing published evidence on the relationship between the community food environment and health outcomes beyond obesity in high income countries. This information will be useful in guiding the development of evidence-based policies and interventions that promote healthier community food environments and enhance public health outcomes.

## Methods

### Designing and Protocol Development

The systematic review was conducted and reported according to the Preferred Reporting Items for Systematic Reviews and Meta-Analyses (PRISMA) **(Supplementary file 1)** [23]. The review protocol was registered in the International Prospective Register of Systematic Reviews database (PROSPERO, CRD42023463886).

### Eligibility Criteria

Studies that reported the association between the community food environment and health outcomes were considered for inclusion. Both subjective (perceived availability, accessibility, and affordability of foods) and objective measures (density, proximity, diversity, and composite index measures of food outlets) of community food environment were considered. Diet-related health outcomes such as diabetes, cardiovascular diseases, and related mortalities were included as outcomes of interest. Given the large number of existing reviews on obesity and overweight outcomes, studies that solely reported on these outcomes were excluded from this review [20, 24]. The review includes both observational and experimental studies that were conducted in high-income countries, as defined by the World Bank [25] **(**Table 1**)**.

**Table 1:** Summary of eligibility criteria

Eligibility characteristics	Inclusion criteria
Study population	Studies involving all age group (children, adults, and elderly)
Interventions/Interest or exposures	• Density, proximity, and diversity of food outlets • Composite measures of food environment such as food desert, retail food environment index, ratio of food outlets, etc.… • Subjective measures such as perceived availability, accessibility, and affordability of foods
Study outcomes	Studies with diet-related noncommunicable diseases outcome such as: • Diabetes mellitus • Hypertension • Cardiovascular diseases • Dyslipidaemia • Insulin resistance • Stroke • Metabolic syndrome • Myocardial infraction • Non-Alcoholic Fatty Liver Disease • Noncommunicable diseases related mortality
Study settings/context	High income countries (as per World bank definition)
Study types	Cohort, cross sectional, and case control studies which includes food environment as predictor of health outcomes
Study duration	Studies until 31 August 2023
Language	Studies available in English language

### Information Sources and Search Strategy

Potential research articles were found by searching electronic bibliographic databases for published work on CINHAL (EBSCOhost), Medline (EBSCOhost), Scopus, Web of Science, Google Scholar, and through reference list searches **(Supplementary file 2)**. The search was limited to publications in English and human subjects**.**

The search terms were classified into four groups: population, intervention/interest, context/settings, and outcome. We employed a combination of Medical Subject Heading (MeSH) keywords, and text words to develop the following search strategy: consumer* OR customer* OR buyer OR people OR child* OR adult* AND food* OR"fast food*"OR"food dispensers, automatic"OR"supermarket*"OR"restaurant*"OR"neighbourhood characteristics"OR"built environment"OR"food deserts"OR"junk food"OR"takeaway food"OR"convenience store"OR cafe OR"food environment"OR"food swamp"AND"diabetes mellitus"OR"hypertension"OR"cardiovascular diseases"OR"noncommunicable diseases"OR"non-alcoholic fatty liver disease"OR"metabolic syndrome"OR"health outcome*"OR"dyslipidaemia*"AND"industrialized countries"OR"industrialised countries"OR"developed nations"OR"industrialized nations"OR"industrialised nations"OR"european union"OR"developed countries"OR"north america"OR"scandinavian and nordic countries"OR"scandinavia"OR"commonwealth"OR"united kingdom"OR"uk"OR"united arab emirates"OR"european union"OR australia OR"new zealand"OR Japan OR Taiwan OR “Hong Kong”.

### Study Selection Process

The studies resulting from the search in all electronic databases were imported into Covidence [26]. Duplicated studies were identified and removed, then articles underwent title and abstract screening by two authors independently, and conflicts were resolved by a third reviewer. Full text screening was conducted by two authors independently, and conflicts were resolved with a third reviewer, where necessary. No studies were excluded on the basis of appraised quality.

### Data Extraction Process and Data Items

Data extraction was completed by one author and a random sample (20%) of studies were checked by another author for correctness and completeness. A data extraction tool was developed a priori based on a comprehensive analysis of previous data extraction templates published in previous reviews. The extracted data included year of publication, country where the study was conducted, community food environment measures, participant characteristics, sample size, health outcomes, and other key findings. The studies were grouped, described, and evaluated in a narrative synthesis as per their methodological and outcome similarities. A variety of diagnostic methods have been considered to define the health outcomes, including self-report, review of medical records, or other diagnostic methods. The secondary outcomes of the review were arthritis and biomarkers of fruit and vegetable intake.

### Study Risk of Bias Assessment

The selected studies were evaluated by one author for the risk of bias assessment using the National Heart, Lung and Blood Institute Quality Assessment tool for Observational Cohort and Cross-Sectional Studies for observational studies [27] and the revised Joanna Briggs Institute (JBI) Critical appraisal (Quality Assessment) tool for quasi experimental studies [28]. The quality tool for observational cohort and cross-sectional studies has 14 questions for critical appraisal of observational studies and finally, the evaluation was classified as good, fair, and poor quality based on the risk of bias that the study methodology introduced. The JBI checklist tool has 9 questions for critical appraisal of quasi-experimental studies, with classifications of high, medium, and low quality.

### Data Synthesis

Due to study heterogeneity, the findings were summarised using a narrative synthesis and presented using tables, graphs and figures. The associations were presented graphically using a harvest plot, created using R software. The reported association were divided into three mutually exclusive categories: no association (“null”), inverse association (“negative”) or positive association (“positive”). Food environment metrics were recategorized based on similarities in food outlet type and dimensions of food environment.

## Results

### Study Selection

**Fig. 1:**
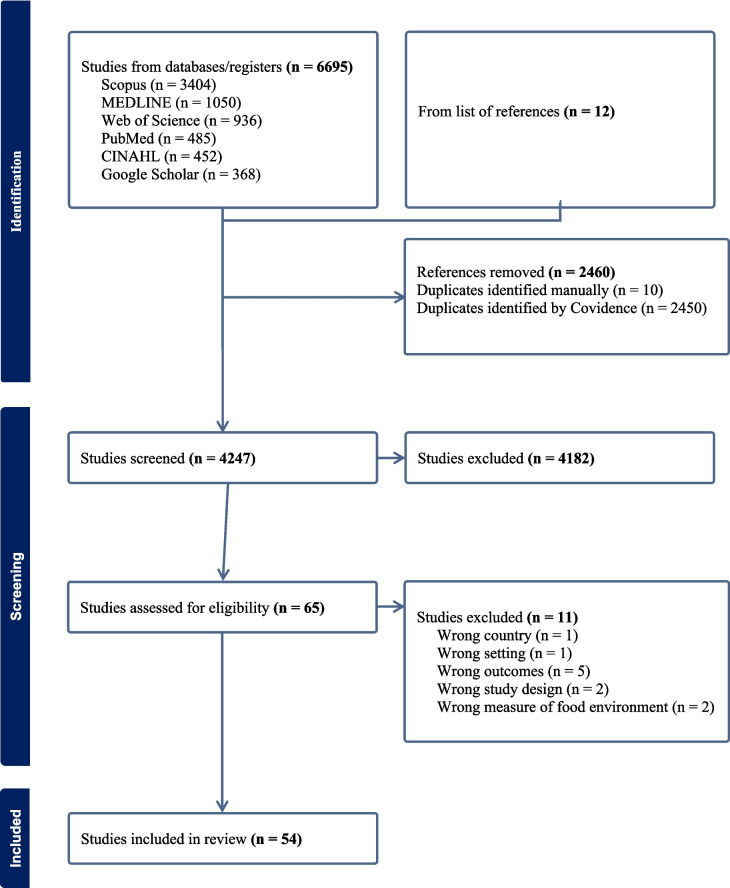
PRISMA flow chart

A total of 6,695 research articles were identified through database searches, and an additional 12 articles were identified through reference list searches. After removal of duplicates and following title and abstract screening, 2,460 and 4,181 articles were removed, respectively. Of the 65 papers reviewed for eligibility, 11 were excluded due to incorrect study setting (n = 2), incorrect outcome (n = 5), incorrect study design (n = 2), and incorrect food environment measurement (n = 2), resulting in 54 articles included in the final review **(**Fig. 1**)**.

### Study Characteristics of Included Articles

The 54 included articles comprised 55 unique studies as one article included three studies, and one study was duplicated in two manuscripts [29–82]. Most of the studies (n = 37; 67.3%) were conducted in the United States, followed by Australia with 5 studies (9.1%). Canada with four studies (7.3%), while the Netherlands, Japan, Sweden, and the United Kingdom contributed two (3.6%) each and the remaining article came from New Zealand (1.8%). A cross-sectional study design was used in 23 (41.8%) studies. The remaining studies used cohort (n = 18; 32.7%), ecological (n = 11; 20%), case–control (n = 2; 3.6%), and quasi-experimental (n = 1; 1.8%) designs **(**Table 2**)**.

**Table 2:** Characteristics of included studies

Authors	Country	Population	Study design	Study quality
(Ntarladima et al., 2022)[29]	Netherland	284, 793 adults older than 19 years	Cross sectional	Good
(Haslam, Nikolaus and Sinclair, 2022)[30]	USA	691 counties	Ecological	Good
(Sheehan et al*.*, 2022) [41]	USA, greater Phoenix metropolitan area, Arizona	133 adolescents aged 14–16 with obesity	Cross sectional	Fair
(Thorpe et al*.,* 2022)[52]	USA, North Carolina, South Carolina, Georgia, Tennessee, Mississippi, Alabama, Louisiana, and Arkansas	11, 208 adults aged 45 or older	Cohort (REGARDS cohort)	Good
(Thorpe et al*.,* 2022)[52]	USA	95, 323 adults with type 2 diabetes as cases and with bout type 23 diabetes as control	Nested case control	Good
(Gondi et al*.,* 2022)[63]	USA	2956 counties	Cross sectional	Good
(Cohen et al*.,* 2021)[74]	USA	1, 402 black adults aged 35–64	Cross sectional	Good
(Lovasi et al*.*, 2021)[79]	USA	2, 753, 000 adults aged 25 years or older	Cohort	Good
(Corona et al*.*, 2021)[80]	USA, Pittsburgh, Pennsylvania	459 women who are black, low income and living in urban	Cross sectional	Good
(Saluja et al*.*, 2021)[81]	Australia, Hunter region, New South Wales (NSW)	3, 070 hospital admitted patients aged 18 years or over	Retrospective Cohort	Good
(Sadler et al*.,* 2021)[82]	USA, Flint, Michigan	160 adults aged 18 or older who were diagnosed with type 2 DM	Cross sectional	Good
(Kanchi et al*.*, 2021)[31]	USA	4, 100, 650 Veteran adults	Retrospective Cohort	Good
(Wiki, Kingham and Campbell, 2021)[32]	New Zealand	167, 272 adults	Ecological	Fair
(Ly et al., 2020)[33]	USA	6006 counties	Ecological	Fair
(Garg et al., 2020) [34]	USA	6, 814 adults aged 45–85 years	Cohort, The Multi-Ethnic Study of Atherosclerosis (MESA)	Good
(Kelli et al*.,* 2019)[35]	USA, Atlanta, and Georgia	4, 944 adults undergone cardiac catheterization	Cohort	Good
(Briggs et al*.*, 2019)[36]	USA, Maine	40, 398 adults aged 18 or over	Cross sectional	Good
(Daniel et al*.*, 2019)[37]	Australia, Adelaide, South Australia	2, 530 adults aged 18 or above	Cohort	Good
(Tani et al*.*, 2019)[38]	Japan	49, 511 older adults aged 65 or over	Cohort	Good
(Baldock et al*.*, 2018)[39]	Australia, northern and western metropolitan regions of Adelaide, South Australia	1491 adults aged 18 or over	Cross sectional	Good
(Poelman et al*.*, 2018)[40]	Netherland	2, 472, 004 adults	Cohort	Fair
(Mazidi and Speakman, 2018)[42]	USA	Adults	Cross sectional	Good
(Liese et al., 2018)[43]	USA, Colorado, and South Carolina	293 youths aged 10–19 years	Case control (Search case control)	Good
(Tabaei et al*.,* 2018)[44]	USA, New York city	182, 756 adults aged 18 or older with diabetes	Cross sectional	Fair
(Sarkar, Webster and Gallacher, 2018)[45]	UK	347, 551 adults aged 37–73	Cross sectional	Good
(Lindsey Haynes-Maslow and Lucia A. Leone, 2017)[46]	USA	3138 Counties	Ecological	Good
(Kelli et al., 2017)[47]	USA, Atlanta metropolitan area	1421 adults aged 20–79 years	Cross sectional	Good
(Richardson et al*.*, 2017)[48]	USA, Pittsburgh Hill/Homewood	831 households	Quasi-experimental	High
(Carrol et al., 2017)[49]	Australia, northern and western regions of Adelaide, South Australia	1945 adults aged 18 or older	Cohort	Good
(Lee et al., 2017)[50]	USA, Massachusetts	4, 010 adults	Longitudinal (Framingham Heart Study)	Fair
(Gebreab et al., 2017)[51]	USA, City of Jackson (Hinds, Rankin, and Madison Counties)	3,6070 African Americans	Cohort (Jackson Heart Study)	Good
(Inoue et al*.*, 2016)[53]	Japan, Chita peninsula, Aichi prefecture	3, 810 elderly aged 65 or older years	Cross sectional	Fair
(Kaiser et al., 2016)[54]	USA, Los Angeles County, California; St. Paul, Minnesota; Chicago, Illinois; Forsyth County, North Carolina, Baltimore, Maryland; and New York, New York	3, 382 adults aged 45–84 years	Cohort, The Multi-Ethnic Study of Atherosclerosis (MESA)	Good
(Alhasan and Eberth, 2016)[55]	USA, South Carolina	46 counties	Ecological	Good
(Herrick, Yount and Eyler, 2016)[56]	USA	15, 522 adults	Cross sectional	Good
(Polsky et al*.*, 2016)[57]	Canada, Toronto, Brampton, Mississauga, and Hamilton	7079 adults aged 20–84 years	Retrospective cohort	Good
(Mezuk et al*.*, 2016)[58]	Sweeden	2, 805, 533 adults aged 35 or older	Cross sectional	Fair
(Frankenfeld, Leslie and Makara, 2015)[59]	USA, Districts of Columbia, Maryland, and Virginia parts of Washington DC metropolitan area	Not stated	Ecological	Fair
(Bodicoat et al., 2015)[60]	UK, Leicestershire	10, 461 adults aged 18–75 years	Cross sectional	Good
(Christine et al., 2015) [61]	USA, (New York, New York; Baltimore, Mary land; Forsyth County, North Carolina; Chicago, Illinois; St Paul, Minnesota, and Los Angeles, California)	5, 124 adults aged 45 to 84 years	Cohort, The Multi-Ethnic Study of Atherosclerosis (MESA)	Good
(Suarez et al*.*, 2015)[62]	USA	22, 173 adults aged 20 years or over	Cross sectional	Fair
(Chum and O’Campo, 2015)[64]	Canada, Toronto	2, 411 adults aged 25–64 years old	Cross sectional	Good
(Paquet et al*.,* 2014)[65]	Australia, Northern and western metropolitan regions of Adelaide, South Australia	3, 145 adults aged 18 or older	Cohort	Good
(Hsieh et al., 2014)[66]	USA, Los Angeles	453 Hispanic Youths aged 8–18	Cross sectional	Fair
(Hamano et al., 2013)[67]	Sweden	2, 115, 974 men and 2, 193, 700 women aged 35–80 years	Cohort	Good
(Salois, 2012)[68]	USA	Counties	Ecological	Poor
(Ahern et al*.*, 2011)[69]	USA	3, 128 adults over 20 years	Ecological	Good
(Daniel et al*.*, 2010)[70]	Canada, Montreal Census Metropolitan Area	826 Census tract	Ecological	Fair
(Morgenstern et al*.*, 2009)[71]	USA, Corpus Christi, Nueces County, Texas	1,247 adults aged 45 or over	Cross sectional	Fair
(Li et al., 2009) [72]	USA, Portland, Oregon	1, 145 adults aged 50 to 75 years	Cohort	Good
(Auchincloss et al., 2009)[73]	USA, Baltimore (city) and Baltimore County, Maryland; Forsyth County, North Carolina, and New York City/Bronx, New York	2, 285 adults aged 45–84 years	Cohort, The Multi-Ethnic Study of Atherosclerosis (MESA)	Good
(Susan H et al*.*, 2008)[75]	USA, California	40, 000 adults aged 18 or older	Ecological	Fair
(Mobley et al*.*, 2006)[76]	USA	2, 134 Women	Cross sectional	Fair
(Morland, Diez Roux and Wing 2006)[77]	USA, Mississippi, North Carolina, Maryland, and Minnesota	10, 763 adults aged 49–73	Cross sectional	Good
(Alter and Eny, 2005)[78]	Canada, Ontario	380 sortation area	Ecological	Fair

### Quality Assessment

Of the 54 observational studies, 68.5% (n = 37), 29.6% (n = 16) and 1.9% (n = 1) were rated as good, fair, and poor quality, respectively. The single quasi-experimental study was rated as high quality **(**Table 2**)**.

### Community Food Environment Measures

In the 55 included studies, 90 different food environment measures were extracted. The food environment measures included: fast-food outlets (n = 28; 31.1%); full serviced restaurant (n = 6; 6.7%); supermarket (n = 7; 7.8%); grocery store (n = 4; 4.4%); fruit and vegetables shop (n = 9; 10%); convenience store (n = 7; 7.8%); farmer’s market (n = 3; 3.3%); “healthy” or “unhealthy” food outlets (n = 17; 18.9%); and liquor selling shop (n = 4; 4.4%). The remaining five food environment measures (5.6%) were composite indices.

Of the 81 food environment measures (90.0%) that were objectively measured, 39 (48.1%) included density of outlets while 12 (14.8%) measured relative density, 19 (23.5%) proximity to the outlets, 9 (11.1%) presence or absence of the outlet and 2 (2.5%) measured market sale.

Of the 90 different food environment measures that were extracted, 63 (70%) focused on food availability, 21(23.3%) on food accessibility, two (2.2%) on food price, one (1.1%) on food affordability, and the remaining three (3.3%) on composite dimensions of the food environment.

### Association of Community Food Environment with Health Outcomes

#### Diabetes, Glycaemic Control, and Insulin Resistance Outcomes

Thirty-four studies measured diabetes as a health outcome, using either prevalence/incidence of diabetes (n = 31) [29–32, 39, 41–43, 45–52, 55–61, 65, 68, 69, 73, 75, 77, 80], glycaemic control (n = 2) [44, 82] and insulin resistance (n = 1) [66]. Most of the studies (n = 29; 85.3%) were conducted in the general adult population, three with adolescents (n = 3; 8.8%) and one in low-income black women living in urban areas **(**Table 3**)**.

**Table 3:** Characteristics of included studies with diabetes, rate of diabetes, glycaemic control, and insulin resistance outcomes

Authers	Food environment measurement	Data source for food environment	Health outcomes	Association between food environment and health outcomes
(Ntarladima et al., 2022)[29]	• Presence of fast-food outlets within 100, 400, 1000 and 1500 m street network buffer • Fast-food outlets density within 400, 1000 and 1500 m street network buffer • Ratio of fast-food outlets within 400, 1000 and 1500 m street network buffer	Commercial database (Locatus, 2014)	• Diabetes	(+) Presence of fast-food outlets within 100, 400, 1000, and 1500 m street network buffer with prevalence of diabetes in urban areas (+) Density of fast-food outlets within 400 m street network buffer with prevalence of diabetes in urban areas (+) Ratio of fast-food outlets within 1000 and 1500 m street network buffer with prevalence of diabetes in urban areas (+) Presence of fast-food outlets within 100, 400, and 1000, m street network buffer with prevalence of diabetes in rural areas (+) Density of fast-food outlets within 400, 1000 and 1500 m street network buffer with prevalence of diabetes in rural areas (+) Ratio of fast-food outlets within 1000 street network buffer with prevalence of diabetes in rural areas
(Haslam, Nikolaus and Sinclair, 2022)[30]	• Low access to grocery stores	USDA food environment Atlas 2020	• Prevalence of diabetes	No association
(Sheehan et al*.*, 2022) [41]	• Lack of supermarket (%) • Number of full-service restaurants per square mile • Number of fast-food restaurants per square mile • Number of aAlcohol drinking places per square mile • Number of snack shops	American community survey, USDA food access research Atlas, 2021, the Arizona healthy community map and the national neighbourhood archive	• Two hours glucose value	(+) Number of fast-food restaurants per square mile with 2-h glucose value (β, 95% CI = 5.48, (2.52, 8,45)) (-) Number of full-service restaurants per square mile with 2-h glucose value (β = −2.00)
(Thorpe et al*.,* 2022)[52]	• Proportion of supermarkets relative to food retail establishments • Proportion of fast-food restaurants relative to food service establishments	The Reasons for Geographic and Racial Differences in Stroke (REGARDS) cohort study	• Type 2 diabetes	No association reported
(Thorpe et al*.,* 2022)[52]	• Proportion of supermarkets relative to food retail establishments • Proportion of fast-food restaurants relative to other food outlets	Geisinger/John Hopkins University	• Type 2 diabetes	(-) Proportion of fast-food restaurants with type 2 diabetes in high density urban area (OR = 0.77, 95% CI (0.67, 0.88))
(Sadler et al*.,* 2021)[82]	• Nutrition Environment Measure Survey in Store Score (NEMS-S)	Flint Food Store Survey (FFSS) 2016	• Glycaemic Control (HbA1 C)	No association reported
(Corona et al*.*, 2021)[80]	• Easy to buy fruits and vegetables (Yes/No) • Large selection of fruits and vegetables (Yes/No) • Access to high quality fruits and vegetables (Accessibility) (Yes/No) • Good price for fruit and vegetable (Affordability) (Yes/No) • Shopping at discount groceries vs full-service groceries • Shopping at supercentres/wholesales vs full-serviced groceries • Speciality stores vs full-service groceries • Price vs quality as of food as reason for shopping • Convenient locations vs quality of foods as a reason for shopping • Other attributes vs quality of foods as reason for shopping • Frequency of shopping at stores with low access to healthy foods	Pittsburgh Hill/Homewood Research on Neighbourhoods and Health (PHRESH) study	• Diabetes	No association reported
(Kanchi et al*.*, 2021)[31]	• Proportion of fast-food restaurants relative to all food service establishments • Proportion of supermarkets related to all food retail establishments	US census tract	• Type 2 diabetes	(+) Proportion of fast-food restaurants relative to all restaurants with risk of type 2 diabetes (HR = 1.01, 95% CI (1.01,1.01)) (-) Proportion of supermarkets relative to all food stores with risk of type 2 diabetes (HR = 0.99, 95% CI (0.99, 0.99))
(Wiki, Kingham and Campbell, 2021)[32]	• Number of fast-food outlets • Number of takeaway outlets • Number of dairy/convenience stores • Number of supermarkets • Number of fruit and vegetable shops	Ministry for Primary Industries and Territorial Authorities (2013–2016)	• Number of Type 2 diabetes	(+) Number of fast-food outlets with number of type 2 diabetes (median = 1.02, 95 CI (1.01, 1.04))) (-) Number of dairy/convenience stores with number type 2 diabetes (median = 0.98, 95 CI (0.96, 0.99)) (-) Number of fruit and vegetable shops with number type 2 diabetes (median = 0.94, 95 CI (0.91,0.96))
(Baldock et al*.*, 2018)[39]	• Perceived distance to fruit and vegetable retailers • Objective distance to fruit and vegetable retailers • Perceived distance overestimates the objective distance to fruit and vegetable shops	2007 South Australia retail database	• Prediabetes/diabetes	No association reported
(Liese et al., 2018)[43]	• Food desert	USDA economic research service 2013	• Type 2 diabetes	No association reported
(Mazidi and Speakman, 2018)[42]	• Density of fast-food restaurants per 1000 population • Density of full-service restaurants per 1000 population	USDA economic research service food environment atlas	• Prevalence of type 2 diabetes	(+) Density of fast-food restaurants per 1000 population with prevalence of type 2 Diabetes (β = 0.76, 95% CI (0.46, 1.06))
(Tabaei et al*.,* 2018)[44]	• Ratio of BMI healthy to BMI neutral and unhealth food outlets • Ratio of BMI neutral to BMI healthy and unhealth food outlets • Ratio of BMI unhealthy to BMI healthy and neutral food outlets	2005 data purchased from Dun & Bradstreet (Short Hills, New Jersey)	• Glycaemic control	(+) The ratio of BMI healthy to BMI neutral and BMI unhealthy food outlets with glycaemic control (OR = 1.09, 95% CI (1.07, 1.11)) (+) The ratio of BMI neutral to BMI healthy and BMI unhealthy food outlets with glycaemic control (OR = 1.50, 95% CI (1.45, 1.54)) (-) The ratio of BMI unhealthy to BMI healthy and BMI neutral food outlets with glycaemic control (OR = 0.75, 95% CI (0.74, 0.77))
(Sarkar, Webster and Gallacher, 2018)[45]	• Density of pubs and bars per km^2^ • Density of restaurants and cafeterias per km^2^ • Density of hot and cold takeaway shops per km^2^ • Composite density of ready to eat food outlets per km^2^ • Street distance to the nearest pubs and bars • Street distance to the nearest restaurants and cafeteria • Street distance to the nearest hot and cold takeaway shops	UK Biobank Urban Morphometric Platform (UKBUMP)	• Type 2 diabetes	(+) Density of restaurants and cafeterias per km^2^ was associated with type 2 diabetes (0.45–1.18 units per km^2^ (OR = 1.09, 95% CI (1.02–1.16)), 1.81–4.76 units per km^2^ (OR = 1.08, 95% CI (1.01–1.15)), > 4.76 units per km^2^ (OR = 1.13, 95% CI (1.05–1.21)) Reference 0 (no exposure) (+) Density of hot and cold takeaway shops per km^2^ was associated with type 2 diabetes (0.75–2.15 units per km^2^ (OR = 1.08, 95% CI (1.01–1.14)), Reference 0 (no exposure) (+) Composite density of ready to use food environments per km^2^ was associated with type 2 diabetes (> 10.7 units per km^2^ (OR = 1.11, 95% CI (1.02–1.21)) Reference 0 (no exposure) (-) Street distance to nearest restaurants and cafeterias with type 2 diabetes (400.8 m-666.7 m street distance, (OR, = 0.9, 95% CI (0.84, 0.97)), (666.7–1002.6 m street distance, (OR = 0.89, 95% CI (0.83, 0.96)) 1002.6 m-1576 m street distance, (OR = 0.86, 95% CI (0.80, 0.93)), > 1576 m street distance, OR = 0.84, 95% CI (0.78, 0.91))). Reference 0–400.8 m (-) Street distance to nearest cold and hot takeaway shops with type 2 diabetes (527.3 m-903.2 m street distance, (OR = 0.92, 95% CI (0.85, 0.99)), (903.2–1434.4 m street distance, (OR = 0.90, 95% CI (0.83, 0.97)) 1434.4 m-2640 m street distance, (OR = 0.93, 95% CI, (0.86, 1.00)), > 2640 m street distance, (OR = 0.91, 95% CI (0.85, 0.99))), Reference 0–527.3 m
(Lindsey Haynes-Maslow and Lucia A. Leone, 2017)[46]	• Density of grocery stores per 1000 residents • Density of supercentres per 1000 residents • Density of farmer’s markets per 1000 residents • Density of fast-food restaurants per 1000 residents • Density of full-service restaurants per 1000 residents • Density of convenience stores per 1000 residents	USDA Economic Research Service, Food Environment Atlas 2012	• Diabetes prevalence	(-) Density of grocery stores per 1000 low poverty low racial composition residents with prevalence of diabetes (β = −0.67, 95% CI (−1.12, −0.22)) (+) Density of fast-food restaurants per 1000 low poverty low racial composition residents with prevalence of diabetes (β = 0.64, 95% CI (0.17, 1.11)) (-) Density of full-service restaurants per 1000 low poverty low racial composition residents with prevalence of diabetes (β = −0.49, 95% CI (−0.74, −0.24)) (-) Density of farmer’s markets per 1000 low poverty medium racial composition residents with prevalence of diabetes (β = −2.50, 95% CI (−3.89, −1.11)) (-) Density of full-service restaurants per 1000 low poverty medium racial composition residents with prevalence of diabetes (β = −0.69, 95% CI (−0.93, −0.45)) (+) Density of convenience stores per 1000 low poverty medium racial composition residents with prevalence of diabetes (β = 1.57, 95% CI (1.21, 1.93)) (-) Density of full-service restaurants per 1000 low poverty high racial composition residents with prevalence of diabetes (β = −1.72, 95% CI (−2.42, −1.01)) (+) Density of convenience stores per 1000 low poverty high racial composition residents with prevalence of diabetes (β = 1.68, 95% CI (0.9, 2.45)) (-) Density of full-service restaurants per 1000 high poverty low racial composition residents with prevalence of diabetes (β = −1.37, 95% CI (−2.15, −0.59)) (-) Density of full-service restaurants per 1000 high poverty medium racial composition residents with prevalence of diabetes (β = −2.00, 95% CI (−2.75, −1.26)) (+) Density of convenience stores per 1000 high poverty medium racial composition residents with prevalence of diabetes (β = 1.63, 95% CI (0.46, 2.80)) (-) Density of full-service restaurants per 1000 high poverty high racial composition residents with prevalence of diabetes (β = −2.25, 95% CI (−3.14, −1.36)) (+) Density of convenience stores per 1000 high poverty high racial composition residents with prevalence of diabetes (β = 1.88, 95% CI (1.17, 2.59))
(Kelli et al., 2017)[47]	• Food desert	USDA Economic Research Service, food access research atlas	• Diabetes • Fasting blood glucose level	(+) Food desert with fasting glucose level
(Richardson et al*.*, 2017)[48]	• Opening supermarket		• High blood sugar • Prevalence of diabetes	There was 3.6% difference on the prevalence of diabetes in intervention as compared to control (no change on the intervention but increased in the control group)
(Carrol et al., 2017)[49]	• Density of fast-food outlets per 1600 m^2^/1 mile • Density of healthful food outlets per 1600 m^2^/1 mile	2007 South Australia retail database	• Cardiometabolic risk/Change in HgA1 C	No association reported
(Lee et al.,2017) [50]	• Number of total food stores per square kilometre • Number of full-service restaurants per square kilometre • Number of fast-food restaurants per square kilometre • Number of supermarkets per square kilometre • Number of convenience stores per square kilometre	2000 US census data (TIGER/Line files)	• Incidence of diabetes • Fasting plasma glucose	(+) Number of fast-food restaurants with fasting plasma glucose (+) Number of supermarkets with fasting plasma glucose
(Gebreab et al., 2017) [51]	• Density of favourable food stores per 1-mile buffer • Density of Unfavourable food stores per 1-mile buffer	National Establishment Time-Series (NETS) database, Walls & Associate for the years 2000–2010 and Nielsen/TDLinx Service Supermarket Retail Category Database (Nielsen Company, 2008)	• Incidence of type 2 diabetes • Prevalent of type 2 diabetes	(+) Density of unfavourable food stores with incidence of type 2 DM (HR = 1.34, 95%CI (1.12,1.60))
(Alhasan and Eberth, 2016)[55]	• Density of fast-food restaurants per 1000 population • Density of convenience stores per 1000 population • Density of super stores per 1000 population • Density of grocery stores per 1000 population	USDA Economic Research Service, food access research atlas	• Prevalence of type 2 diabetes	No association observed
(Herrick, Yount and Eyler, 2016)[56]	• Density of supermarkets per square mile		• Risk of diabetes	(-) Density of supermarkets per square mile with high risk of diabetes (AOR = 0.84, 95% CI (0.71, 0.99))
(Polsky et al*.*, 2016)[57]	• Number of fast-food restaurants within 10 min walking distance (720 m) • Proportion of fast-food restaurants relative to all restaurants within 10 min walking distance (720 m)	Purchased from Dun and Bradstreet, Canada 2008	• Incidence of diabetes	No association reported
(Mezuk et al*.*, 2016)[58]	• Ratio of health harming food outlets to total food outlets per km^2^		• Prevalence and incidence of type 2 diabetes	(+) Ratio of health harming food outlets to total food outlets with prevalence of type 2 diabetes (AOR = 1.88, 95% CI (1.51, 2.26)) (+) Ratio of health harming food outlets to total food outlets with incidence of type 2 diabetes (AOR = 2.11, 95% CI (1.57, 2.82)) (+) Staying at high ratio of health harming food outlets to total food outlets with incidence of type 2 diabetes (AOR = 1.72, 95% CI (1.27, 2.33)) (+) Moving to high ratio of health harming food outlets to total food outlets with incidence of type 2 diabetes (AOR = 3.67, 95% CI (2.14, 6.30))
(Christine et al., 2015)[61]	• Number of supermarkets and/or fruit and vegetable shops per mile square • Perceived availability of healthy foods within 1 mile distance or 20-min walk	Annual information from the National Establishment Time Series database for the years 2000 through 2012 and community survey	• Incidence of type 2 diabetes mellitus	(-) Perceived availability of healthy food within 1 mile distance with incidence of type 2 diabetes mellitus (HR = 0.88, 95%CI (0.78, 0.98))
(Frankenfeld, Leslie and Makara, 2015)[59]	• Retail food environment (RFE) used to create two categories, “healthier” and “unhealthier” options • The healthier options subdivided to: ✓Grocery store ✓Restaurants, and ✓Speciality store • The unhealthier options subdivided to: ✓Convenient store and (check) Restaurants and fast food	Dun & Bradstreet’s Hoovers	• Prevalence of diabetes • Fruit and vegetable intake	There were significant differences in the prevalence of Diabetes and fruit and vegetable intake across the sub-types within the healthier options category; healthier options category, the sub-types with higher restaurants and higher specialty food had lower diabetes prevalence than the grocery stores sub-type
(Bodicoat et al., 2015)[60]	• Number of fast-food outlets within 500 m radius	Online business listing (Thompson’s directory)	• Type 2 Diabetes • Impaired glucose regulation • Fasting glucose mmol/l • HbA1c (%)	(+) Number of Fast-food outlets with in 500 m radius with type 2 Diabetes (AOR = 1.02, 95% CI (1.00,1.04))
(Hsieh et al., 2014)[66]	• Number of fast-food restaurants at 0.5-mile buffer • Number of fast-food restaurants at 1 mile buffer • Number of fast-food restaurants at 2-mile buffer • Number of convenient stores at 0.5-mile buffer • Number of convenient stores at 1-mile buffer • Number of convenient stores at 2-mile buffer	InfoUSA	• Insulin Resistance (IR) defined by HOMA score	(+) Number of fast-food restaurants at 1 mile walking distance with insulin resistance among boys (β-Coefficient = 0.06)
(Paquet et al*.,* 2014)[65]	• Relative Food Environment Index (RFEI) (the ratio of unhealthful to healthful food sources)	1999 South Australia Retail Database	Pre-diabetes/diabetes	No association reported
(Salois, 2012)[68]	• Density of fast-food restaurants per 1000 population • Density of full-service restaurants per 1000 population • Density of supermarkets/grocery stores per 1000 population • Density of convenience stores with no gas per 1000 population • Density of convenience stores with gas per 1000 population • Density of supercentre and club stores per 1000 population • Percent of farms with direct sale • Direct farm sale per capita (dollars) • Density of farmer’s markets per 1000 population	USDA Economic Research Service, food access research atlas	• Prevalence of diabetes	(+) Density of fast-food restaurants per 1000 population with prevalence of diabetes (+) Density of gas-based convenience stores with prevalence of diabetes (+) Density of non-gas-based convenience stores per 1000 population with prevalence of diabetes (-) Local food economy with prevalence of diabetes and obesity (-) Direct farm sale per capita (dollars) with rate of obesity and diabetes (A $100 increase in per capita direct farm sale is associated with 0.80% lower obesity rate and a 1.2% lower diabetes rate) (-) Density of farmer’s markets with rate of diabetes (Every additional farmers’ market per 1000 people is associated with a 0.78% lower diabetes rate)
(Ahern et al*.*, 2011)[69]	• Number of fast-food restaurants per 1, 000 population • Number of full-service restaurants per 1, 000 population • Number of grocery stores per 1, 000 population • Number of convenience stores per 1, 000 population • Direct $ farm sales per capita • % of households with no car living more than one mile from grocery stores	USDA Economic Research Service, food access research atlas and 2007 agricultural census	• Rate of adult diabetes (obtained from CDC Behavioural Risk Factor Surveillance System (BRESS) survey and US census estimate for the year 2006–2008)	(+) Number of fast-food restaurants per 1, 000 population with rate of diabetes (β-Coefficient = 0.41) (+) Number of convenience stores per 1000 population rate of diabetes (β-Coefficient = 0.30) (+) % of households with no car living more than one mile from grocery stores with rate of diabetes (β-Coefficient = 0.07) (-) Number of full-service restaurants per 1000 population with rate of diabetes (β-Coefficient = −0.15) (-) Number of grocery stores per 1000 population with rate of diabetes (β-Coefficient = −0.37)
(Auchincloss et al., 2009)[73]	• Perceived availability of healthy foods within 1 mile distance from home or 20-min walk	Community survey	• Incidence of type 2 diabetes	(-) Perceived availability of healthy foods with incidence of diabetes (HR = 0.63, 95%CI (0.42, 0.93)
(Susan H et al*.*, 2008)[75]	• RFEI at 0.5 mile in urban, 1-mile smaller cities and 5 miles in rural	2005 InfoUSA business file	• Prevalence of diabetes	(+) RFEI with prevalence of diabetes
(Morland, Diez Roux and Wing 2006)[77]	• Number of supermarkets • Number of grocery stores and • Number of convenience stores • Number of full-service restaurants • Number fast-food outlets	1999 local; department of environmental and state department of agriculture	• Prevalence rate of Diabetes	No association observed

**Fig. 2:**
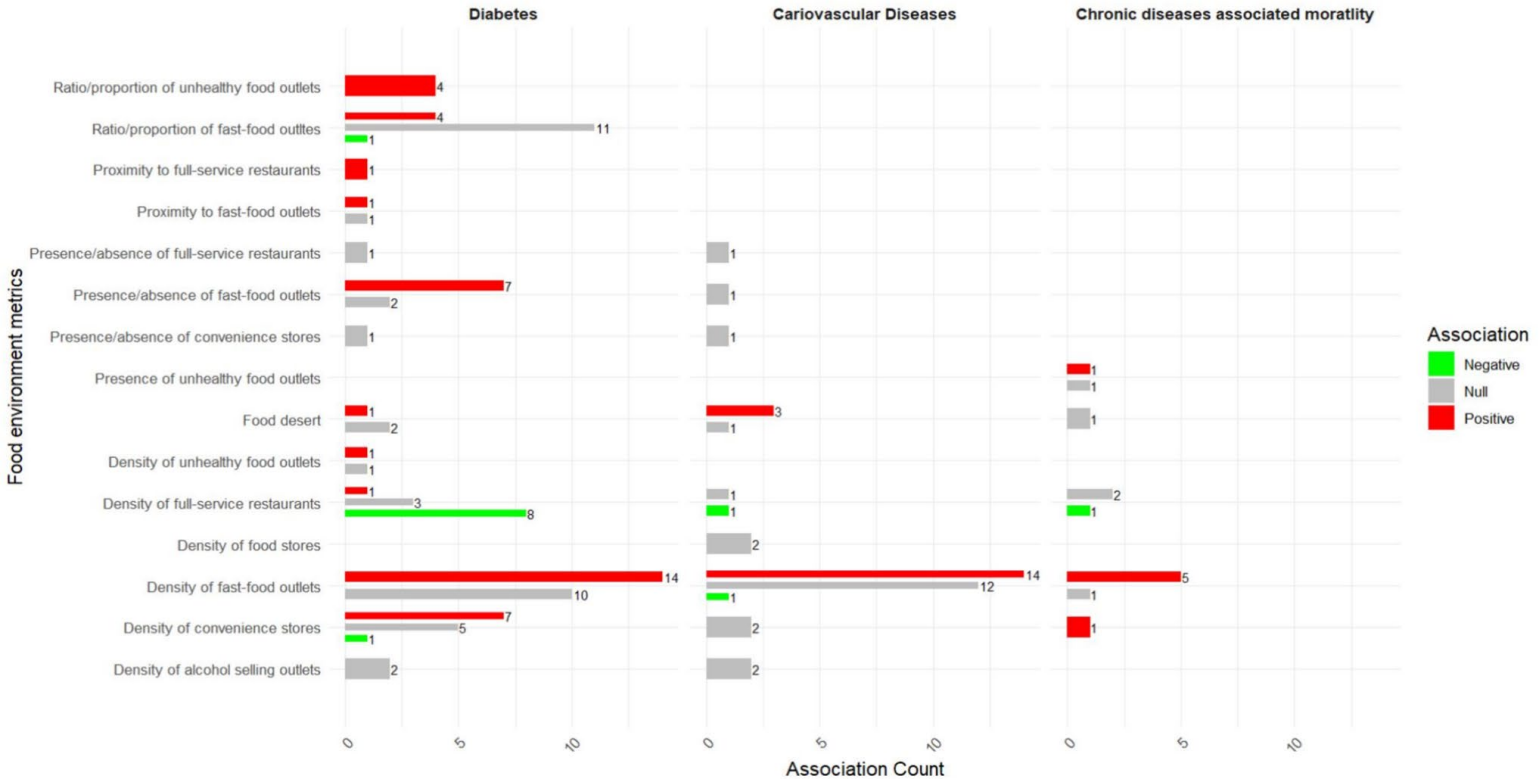
Harvest plot of the association between food environment metrics derived from unhealthy and less healthy food outlets and health outcomes

**Fig. 3:**
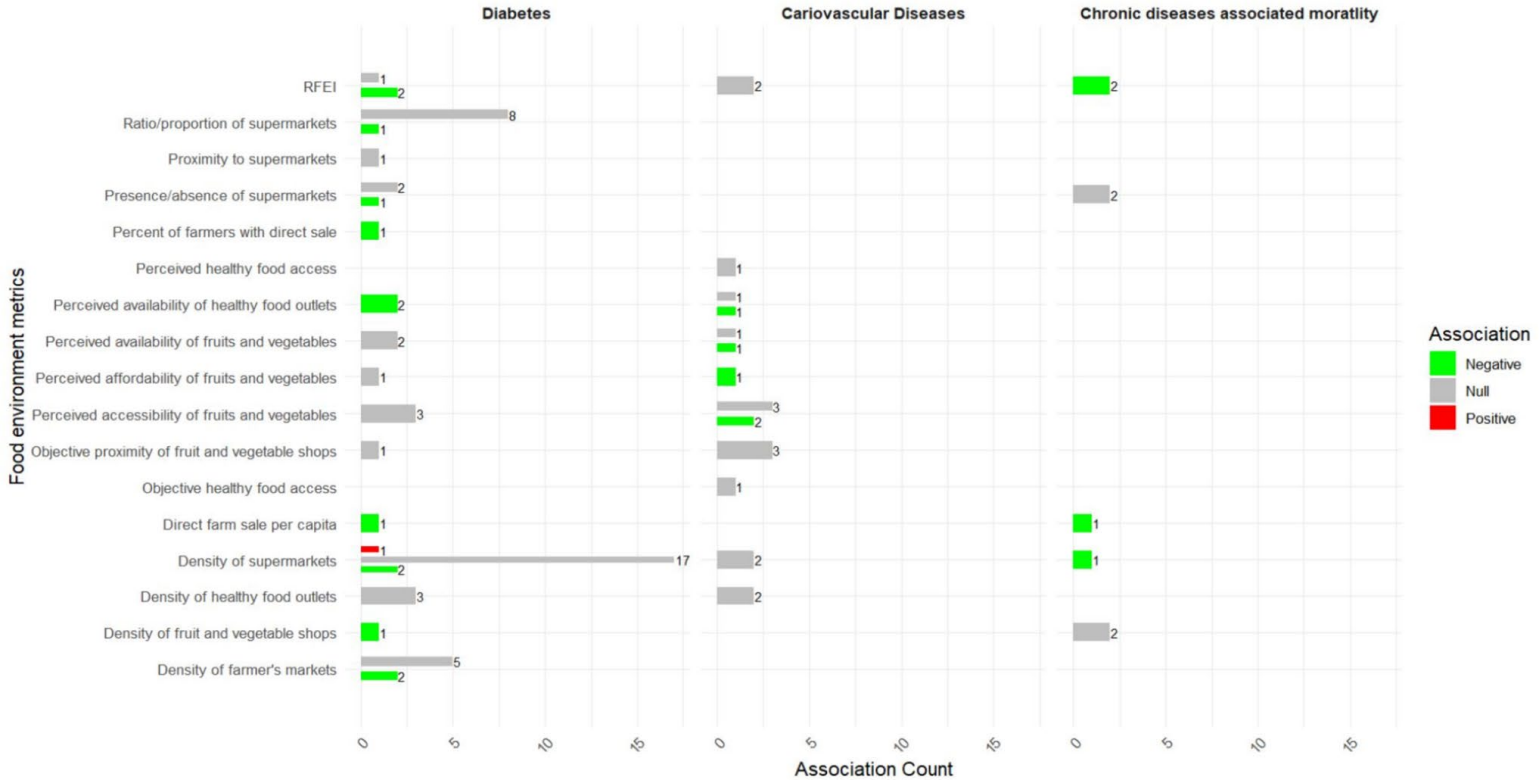
Harvest plot of the association between food environment metrics derived from healthy food outlets and health outcomes

Of the 31 studies that examined the association between the community food environment and diabetes, 21 (67.7%) reported at least one significant association. One hundred and forty-eight associations were extracted from the final models involving food environment measures and diabetes, of which 43.9% reported significant associations (n = 65/148). Food environment metrics related to unhealthy food outlets **(**Fig. 2**)**, such as the density of fast-food outlets (n = 14/24), density of convenience stores (n = 7/13), presence of fast-food outlets (n = 7/9), and ratio/proportion of unhealthy food outlets (n = 4/4) were predominantly positively associated with diabetes, with higher concentration of unhealthy food outlets associated to increased diabetes outcomes. Conversely, food environment metrics related to less healthy food outlets (Fig. 2) and healthy food outlets **(**Fig. 3**)**, including the density of full-service restaurants (n = 8/11), Retail Food Environment Index (RFEI) (n = 2/3), perceived availability of healthy food outlets (n = 2/2), density of fruit and vegetable shops (n = 1/1), percentage of farmers with direct sales (n = 1/1), and direct farm sales per capita (n = 1/1) showed predominantly negative significant associations.

Food environment metrics related to healthy food outlets **(**Fig. 3**)**, such as the density of supermarkets (n = 17/20), ratio/proportion of supermarkets (n = 8/9), presence/absence of supermarkets (n = 2/3), proximity to supermarkets (n = 1/1), density of farmers’ markets (n = 5/7), density of healthy food outlets (n = 3/3), perceived availability (n = 2/2), accessibility (n = 3/3), and affordability (n = 1/1) of fruit and vegetable shops, as well as objective proximity to fruit and vegetable shops, were predominantly not associated with diabetes. Similarly, food environment metrics related to less healthy and unhealthy food outlets **(**Fig. 2**)**, such as the ratio/proportion of fast-food outlets (n = 11/16), density of alcohol selling outlets (n = 2/2), food deserts (n = 2/3), presence/absence of convenience stores (n = 1/1), and presence/absence of full-service restaurants (n = 1/1), were also not associated.

Four food environment measures were used in studies that reported glycaemic control as the outcome measure. The ratio of healthy to neutral/unhealthy food outlets and the ratio of neutral to healthy/unhealthy food outlets were positively associated with good glycaemic control whereas unhealthy to healthy/neutral food outlets was negatively associated with good glycaemic control [44]. The study that used the Nutrition Environment Measure Survey in Store Score (NEMS-S), reported a null association with glycaemic control [82] **(**Table 3**)**.

Six food environment measures were used in the studies that included insulin resistance as the outcome, namely: number of fast-food outlets within 0.5, 1, and 2-miles buffer and number of convenience store within 0.5, 1 and 2-miles buffer. Of the 12 associations, when stratified by sex, 91.7% (n = 11/12) reported a null association. The number of fast-food outlets at 1 mile walking distance from residential location among boys was positively associated with insulin resistance [66] **(**Table 3**)**.

#### Cardiovascular Diseases and Mortalities and Other Outcomes

Cardiovascular diseases [36, 37, 40, 47, 48, 53, 64, 74, 76], coronary heart diseases [40, 78], myocardial infarction [35, 64, 81], and heart failure [40], hypertension [39, 47, 48, 54, 62, 65, 72, 77, 80], stroke [40, 67, 71], and atrial fibrillation [34] were all included as health outcomes in 22 studies **(**Table 4**)**.

**Table 4:** Characteristics of included studies with cardiovascular diseases, mortalities, and other health outcomes

Authers	Food environment measurement	Data source for food environment	Health outcomes	Association between food environment and health outcome
(Gondi et al*.,* 2022)[63]	• Food Environment Index (FEI) and Food insecurity (FI%)	2015 USDA economic research service food environment atlas	• Heart Failure mortality (HF MR)	(-) Food environment index with HF MR (β = − 3.6% per 1-unit increase in FEI) (+) Food insecurity % with HF MR (β = − 1.3% per 1% decrease)
(Cohen et al*.,* 2021)[74]	• Perceived neighbourhood healthy foods access • Objectively measured healthy foods access	2015 USDA economic research service food environment atlas	• Cardiovascular diseases	No association
(Lovasi et al*.*, 2021)[79]	• Presence of supermarkets/produce markets (healthy food retails) • Presence of unhealthy food retails	National Establishment Time Series (NETS) data	• Cardiovascular mortality • All causes mortality	(+) Presence of unhealthy food retails with all causes of mortality (HR = 1.15, 95%CI (1.11, 1.20))
(Corona et al*.*, 2021)[80]	• Easy to buy fruits and vegetables (Yes/No) • Large selection of fruits and vegetables (Yes/No) • Access to high quality fruits and vegetables (Accessibility) (Yes/No) • Good price for fruits and vegetables (Affordability) (Yes/No) • Shopping at discount grocery vs full-service grocery • Shopping at supercentre/wholesale club vs full serviced grocery • Speciality store vs full-service grocery • Price vs quality as of food as reason for shopping • Convenient location vs quality of foods as a reason for shopping • Other attributes vs quality of food as reason for shopping • Frequency of shopping at stores with low access to healthy foods	Pittsburgh Hill/Homewood Research on Neighbourhoods and Health (PHRESH) study	• Hypertension • Cholesterol level • HDL level • Self-rated health	(-) Easy to buy fruits and vegetables was associated with blood pressure (AOR = 0.47; 95% CI (0.28, 0.79)) and poor/fair self-rated health (AOR = 0.59; 95%CI (0.39, 0.90)) (-) Perceived fruits and vegetables affordability (good price) with blood pressure (AOR = 0.59; 95%CI (0.36, 0.96)) and poor/fair self-rated health (AOR = 0.64; 95%CI (0.42, 0.97)) (-) Perceived access to high quality fruits and vegetables, and poor/fair self-rated health (AOR = 0.62; 95%CI (0.41, 0.94)) (-) Frequent visit to a primary store with low HDL (AOR = 0.74; 95%CI (0.55, 0.98)) (+) Choosing primary food store based on price with high cholesterol (AOR = 2.02; 95%CI (1.19, 3.45)) (+) Shopping often at stores with low access to healthy foods with high cholesterol (AOR = 3.52; 95%CI (1.09, 11.40)) (-) Shopping often and sometimes as compared with rarely at stores with high access to healthy foods with poor/fair self-rated health (AOR = 0.36; 95%CI (0.15, 0.85) and AOR = 0.58; 95%CI (0.36, 0.92))
(Saluja et al*.*, 2021)[81]	• Density of fast-food outlets per 100, 000 population	Local government councils’ registration and Google maps	• Myocardial Infraction (MI) from Hunter Cardiac and Stroke Out Come Unit	(+) Density of fast-food outlets with MI (β coefficient = 4.07; 95%CI (3.86, 4.28)) (An increase of one fast-food outlet per 100 000 people in an LGA corresponded with four additional cases of MI (P < 0.001))
(Ly et al., 2020)[33]	• Food Environment Index (FEI) done by confirmatory factor analysis	United States Department of Agriculture (USDA) Food Environment Atlas, Atlas of Heart Disease and Stroke from the Centres for Disease Control and Prevention (CDC), and Health Rankings from County Health Rankings & Roadmaps (CHR)	• Cardiovascular disease mortality rate	(-) FEI with Cardiovascular disease mortality rate (Coefficient (standard error), −2.02 (0.19)) (a one-point increase in FEI is associated with a decrease of 2.57 (z ≤ 0.05) CVD deaths per 100,000 population)
(Garg et al., 2020) [34]	• Density of healthy food stores within 1 mile^2^ of participants home address • Perceived availability of healthy foods	Dun and Bradstreet data	• Incident atrial fibrillation	No association
(Kelli et al*.,* 2019)[35]	• Food desert (FD)	2015 USDA Food Access Research Atlas	• Myocardial Infraction (MI) • MI associated death	(+) food desert with MI (HR = 1.44; 95%CI (1.06, 1.96)) (+) low income with MI and MI associated death (HR = 1.39; 95%CI (1.06, 1.83) and HR = 1.18; (1.03, 1.35))
(Briggs et al*.*, 2019)[36]	• Density of fast-food restaurants per 1000 population • Density of convenience stores per 1000 population • Density of full-service restaurants per 1000 population • Density of grocery stores per 1000 population	2010–2012 USDA economic research service food environment atlas	• Cardiovascular Health (CVH)	(+) low density full-serviced restaurants with poor CVH score (AOR = 1.38; 95% CI (1.19–1.59))
(Daniel et al*.*, 2019)[37]	• Density fast-food outlets per 1 mile road distance • Number of fast-food outlets per 1 mile road distance • Retail food environment index (RFEI) per 1 mile road distance	1999 South Australia retail data base	• Cardiometabolic risk score (Northwest Adelaide Health Study (NWASH))	No association observed
(Tani et al*.*, 2019)[38]	• Density of healthy food stores within 500 m • Density of healthy food stores within 1000 m • Subjective availability of healthy food • Objective availability of restaurants with in 500 m • Objective availability of convenience store within 500 m • Presence of community centres within 500 m	Ministry of economic, trade and industry commerce establishment survey 2007	Incidence of dementia	(-) Objective availability of food stores within 500 m with incidence of dementia (quartile 3 HR = 1.19; 95%CI (1.06, 1.34) and quartile 2 HR = 1.20; 95%CI (1.07, 1.35) reference quartile 4 (highest availability)) (-) Objective availability of food stores within 1000 m with incidence of dementia (quartile 3 HR = 1.34; 95%CI (1.18, 1.53) and quartile 2 HR = 1.41; 95%CI (1.23, 1.61) reference quartile 4 (highest availability)) (-) Subjective availability of food stores with incidence of dementia (Middle low HR = 1.19; 95%CI (1.04, 1.35) and lowest HR = 1.30 (1.10, 1.52) reference highest subjective availability) (+) Objective availability of restaurants within 500 m with incidence of dementia (quartile 3 HR = 0.84; 95%CI (0.72, 0.97))
(Baldock et al*.*, 2018)[39]	• Perceived distance to fruits and vegetables retailers • Objective distance to fruits and vegetables retailers • Perceived distance overestimates the objective distance to fruits and vegetables shops	2007 South Australia retail database	• Metabolic syndrome, • Hypertension, • Dyslipidaemia	(+) Perceived distance to fruits and vegetables retails with hypertension (OR = 1.19; 95%CI (1.05, 1.34)) (+) perceived distance overestimates the objective distance to fruits and vegetables shops with hypertension (OR = 1.36; 95%CI (1.02, 1.80))
(Poelman et al*.*, 2018)[40]	• Density of fast-food outlets within 500 m • Density of fast-food outlets within 1000 m • Density of fast-food outlets within 3000 m		Cardiovascular diseases (CVD), coronary heart diseases (CHD), Strok, and Heart Failure (HF)	(+) Density of fast-food outlets within 500 m with incidence of cardiovascular diseases in urban area (1 fast-food restaurant AOR = 1.05; 95%CI (1.02,1.08), 2 fast-food restaurants AOR = 1.07; (1.04, 1.12), 3 or more fast-food restaurants AOR = 1.04; 95%CI (1.01, 1.07) reference no fast-food restaurant) (+) Density of fast-food outlets within 500 m with incidence of coronary heart disease in urban area (1 fast-food restaurant AOR = 1.11; 95%CI (1.05,1.17), 2 fast-food restaurants AOR = 1.13; (1.05, 1.21), 3 or more fast-food restaurants AOR = 1.08; 95%CI (1.02, 1.14) reference no fast-food restaurant) (+) Density of fast-food outlets within 500 m with incidence of stroke in urban area (3 or more fast-food restaurants AOR = 1.09; 95%CI (1.02, 1.17) reference no fast-food restaurant) (+) Density of fast-food outlets within 500 m with incidence of heart failure in urban area (2 fast-food restaurants AOR = 1.15; 95%CI (1.03, 1.27) reference no fast-food restaurant) (+) Density of fast-food outlets within 1000 m with cardiovascular diseases in urban area (2 to 4 fast-food restaurants AOR = 1.04; 95%CI (1.00,1.07), 5 or more fast-food restaurants AOR = 1.05; (1.02, 1.09), reference no fast-food restaurant) (+) Density of fast-food outlets within 1000 m with coronary heart disease in urban area (2 to 4 fast-food restaurants AOR = 1.09; 95%CI (1.02,1.17), 5 or more fast-food restaurants AOR = 1.17; (1.09, 1.25), reference no fast-food restaurant) (+) Density of fast-food outlets within 1000 m with heart failure in urban area (5 or more fast-food restaurants AOR = 1.18; (1.05, 1.33), reference no fast-food restaurant) (+) Density of fast-food outlets within 500 m with coronary heart disease in rural area (1 fast-food restaurant AOR = 1.09; 95%CI (1.01,1.17), 2 fast-food-restaurants AOR = 1.20; (1.09, 1.32) reference no fast-food restaurant) (+) Density of fast-food outlets within 500 m with heart failure in rural area (3 or more fast-food restaurants AOR = 1.25; 95%CI (1.01,1.56) reference no fast-food restaurant)
(Mazidi and Speakman, 2018)[42]	• Density of fast-food restaurants per 1000 population • Density of full-service restaurants per 1000 population	USDA economic research service food environment atlas	• Mortality from CVD • Mortality from stroke	(+) Density of fast-food restaurants per 1000 population with CVD mortality (β = 1.10; 95% CI (0.80–1.40)) (+) Density of fast-food restaurants per 1000 population with stroke mortality (β = 0.89 95% CI (0.58–1.19))
(Kelli et al., 2017)[47]	• Food desert	USDA Economic Research Service, food access research atlas	• Hypertension • Hyperlipidaemia • Atherosclerotic cardiovascular diseases (ASCVD) • Inflammatory markers (0xidative stress markers • Valvular function (Augmentation index)	(+) Food desert with hypertension (+) Food desert with higher Atherosclerotic cardiovascular diseases (ASCVD) score (+) Food desert with inflammatory markers and oxidative stress markers (Glutathione) (-) Food desert with valvular function (Augmentation index)
(Richardson et al*.*, 2017)[48]	• Opening supermarket		• Heart disease • High cholesterol • Hypertension • Arthritis	• High cholesterol was observed in the control group • Significant difference in the arthritis among the intervention and control group
(Inoue et al*.*, 2016)[53]	• Perceived proximity to shops selling fruits and vegetables • Objective proximity to shops selling fruits and vegetables		• Cardiovascular risk (Suita Score)	No association observed
(Kaiser et al., 2016)[54]	• Density of favourable food stores within 1 mile square • Perceived availability of healthy foods	Community survey and commercially available business listings through the National Establishment Time-Series database (Walls and Associates, Oakland, California)	• Incidence of hypertension	(-) Perceived availability of healthy food stores with incidence of hypertension (HR = 0.9, 95%CI (0.83, 0.97))
(Suarez et al*.*, 2015)[62]	• Food Desert	USDA Economic Research Service, food access research atlas	• Chronic kidney diseases (CKD) • Hypertension • Serum carotenoid level (a biomarker for fruit and vegetable intake)	(+) Foos desert with systolic blood pressure (β-coefficient (95%CI) = 1.53, (0.41, 2.66)) (-) FD with serum carotenoid level (a biomarker for fruit and vegetable intake)
(Chum and O’Campo, 2015)[64]	• Density of food stores per square kilo metre • Density of fast-food outlets per square kilo metre	City of Toronto Public Health food inspection reports	• Myocardial infraction • Cardiovascular diseases (Self-report)	No association reported in complete adjusted model
(Paquet et al*.,* 2014)[65]	• Relative Food Environment Index (RFEI) (the ratio of unhealthful to healthful food sources)	1999 South Australia Retail Database	• Hypertension, • Dyslipidaemia	No association reported
(Hamano et al., 2013)[67]	• Number of fast-food restaurants per Small Area Market Statistics, SAMS) • Number of bars/pubs per SAMS	Teleadress 2005	• Stroke	(+) Number of fast-food restaurants with stroke among men and women ((OR = 1.02, 95%CI (1.00, 1.05)) for men and (OR = 1.03, (1.00, 1.06)) for women)
(Ahern et al*.*, 2011)[83]	• Number of fast-food restaurants per 1, 000 population • Number of full-service restaurants per 1, 000 population • Number of grocery stores per 1, 000 population • Number of convenience stores per 1, 000 population • Direct $ farm sales per capita • Percentage of households with no car living more than one mile from grocery store	USDA Economic Research Service, food access research atlas and 2007 agricultural census	• Rate of age adjusted mortality • Rate of adult obesity (obtained from CDC Behavioural Risk Factor Surveillance System (BRESS) survey and US census	(+) Number of fast-food restaurants per 1000 population with rate of age adjusted mortality (β-Coefficient = 16.30) (+) Number of convenience stores per 1000 population with rate of age adjusted mortality (β-Coefficient = 16.17) (-) Number of full-service restaurants per 1000 population with rate age adjusted mortality (β-Coefficient = −20.98) (-) Number of grocery stores per 1000 population with rate of age adjusted mortality (β-Coefficient = −28.73) (-) Direct $ farm sales per capita with age adjusted mortality (β-Coefficient = −0.49)
(Daniel et al*.*, 2010)[70]	• Density of fast-food restaurants per square kilometre • Density of fruits and vegetable stores per square kilometres	Montreal Census Metropolitan Area (MCMS) 2001	• Cardiovascular Mortality • Other causes mortality	(+) Density of fast-food restaurants per square kilometre with cardiovascular mortality (RR = 1.39; 95%CI (1.19, 1.63)) (+) Density of fast-food restaurants per square kilometre with other causes mortality (RR = 1.36; 95%CI (1.18, 1.57))
(Morgenstern et al*.*, 2009)[71]	• Number of fast-food restaurants per 1 mile buffer		• Ischemic stroke	(+) Number of fast-food restaurants per 1-mile buffer with ischemic stroke (RR, 95% CI = 1.13, (1.02, 1.25))
(Li et al., 2009) [72]	• Density of fast-food restaurants per mile square • Weekly visit to local fast-food restaurants	Commercially purchased business data from http://infousa.com	• Blood pressure	(+) Density of fast-food restaurants with systolic blood pressure in low walkability neighbourhood (B coefficient = 8.6) (+) Density of fast-food restaurants with diastolic blood pressure in low walkability neighbourhood (B coefficient = 4.0) (+) Visiting fast-food restaurants 1–2 times or more each week with systolic blood pressure in low walkability neighbourhood (B coefficient = 3.1) (+) Visiting fast-food restaurants 1–2 times or more each week with diastolic blood pressure in low walkability neighbourhood (B coefficient = 1.94)
(Mobley et al*.*, 2006)[76]	• Number of full-sized grocery stores per 1000 residents • Number of fast-food outlets per 1000 residents • Number of restaurants per 1000 residents • Number of minimarts per 1000 residents		• Log of CHD	No association observed
(Morland, Diez Roux and Wing 2006)[77]	• Number of supermarkets • Number of grocery stores • Number of convenience stores • Number of fast-food restaurants • Number of full-service restaurants	1999 local departments of environmental health and state departments of agriculture	• High cholesterol • Hypertension	No association reported
(Alter and Eny, 2005)[78]	• Number of fast-food outlets per 100, 000 population		• Per capita mortality rate • Per capita acute coronary syndrome hospitalization rate	(+) Number of fast-food outlets per 100,000 population with Mortality (AOR; 95%CI = 2.52 (1.54, 4.13)) (+) Number of fast-food outlets per 100,000 population with acute coronary syndromes hospital admission (AOR = 2.62; 95%CI (1.42, 3.59))

Of the 22 studies that analysed the association between food environment measures and cardiovascular diseases, 12 (54.5%) reported at least one significant association. Of the 67 examined associations between food environment metrics and cardiovascular diseases, 35.8% (n = 24/67) were significantly associated. The density of fast-food outlets (n = 14/27) and living in food deserts showed predominantly positive significant associations with cardiovascular diseases **(**Fig. 2**)**. Conversely, perceived affordability of fruit and vegetable shops (n = 1/1) showed a negative association **(**Fig. 3**)**.

Food environment metrics related to healthy food outlets **(**Fig. 3**)**, such as the presence/absence of supermarkets (n = 4/4), perceived healthy food access (n = 1/1), perceived accessibility of fruit and vegetable shops (n = 3/5), objective proximity of fruit and vegetable shops (n = 3/3), objective healthy food access (n = 1/1), density of supermarkets (n = 2/2), density of healthy food outlets (n = 2/2), and RFEI (n = 2/2), and food environment metrics related to less healthy and unhealthy food outlets **(**Fig. 2**)**, such as presence/absence of full-service restaurants (n = 1/1), presence/absence of fast-food outlets (n = 1/1), presence/absence of convenience store (n = 1/1), density of food stores (n = 2/2), density of convenience stores (n = 2/2), and density of alcohol selling outlets (n = 2/2), were predominantly not associated (null) with cardiovascular diseases.

Eight studies that included mortality as a health outcome (all-cause mortality [69, 70, 78, 79], cardiovascular disease mortality [33, 42, 70, 79], heart failure mortality [63], myocardial infraction mortality [35], and stroke mortality [42]) included 10 different food environment measures **(**Table 4**)**. Of the 8 studies that examined the association between food environment and mortality, 7 (87.5%) reported at least one significant association. Of the 21 analyses conducted between food environment measures and mortality, 61.9% (13/21) were significantly associated. Mortality was predominantly positively associated with the density of fast-food outlets (n = 5/6) and density of convenience stores (n = 1/1) **(**Fig. 2**)** whereas it was predominantly negatively associated with the density of supermarket (n = 1/1), direct farm sale per capita (n = 1/1), and RFEI (n = 2/2) **(**Fig. 3**)**.

Dyslipidaemia was included as a health outcome in 6 studies [39, 47, 48, 65, 77, 80]. Of six studies, only one reported a significant association between the food environment and dyslipidaemia. Of 19 extracted associations between food environment measures and dyslipidaemia, 5.3% (1/19) were significantly associated. Dyslipidaemia was negatively associated with the opening of a supermarket [48] in a food desert area **(**Table 4**)**.

Self-rated health [80] (n = 4 different food environment measures), composite cardiometabolic risk score) [37] (n = 3), incidence of dementia [38] (n = 6), metabolic syndrome [39] (n = 3), arthritis [48] (n = 1), and chronic kidney diseases [62] (n = 1) were included in one study as health outcomes. Three of the four associations (75%) that examined the association between food environment measures and poor self-rated health significantly and negatively associated with perceptions it being easy to buy fruits and vegetables, perceived affordability of fruits and vegetables, and perceived access to high quality fruits [80]. Of the 7 examined associations between food environment measures and incidence of dementia, 57.1% (4/7) were significantly associated. Objective availability of food store within 500 m or 1000 m and subjective availability of food stores were negatively associated with incidence of dementia whereas objective availability of a restaurant within 500 m was positively associated with incidence of dementia [38] **(**Table 4**)**.

#### Secondary Outcome

Among the 55 included studies, 4 reported at least one secondary outcome. Biomarkers of oxidative stress and inflammation [47], fruit and vegetable intake [59], arthritis [48], and serum carotenoid levels [62] were mentioned in one study each. Living in food desert areas was associated with higher levels of inflammatory and oxidative stress indicators [47] and lower levels of serum carotenoid (biomarkers of fruit and vegetable intake) [62]. Introducing a supermarket in a food desert area was negatively associated with rate of arthritis[48] **(**Table 4**)**.

### Associations by Study Design and Study Quality

Seventeen studies reported no association between community food environment and health outcomes. Of these, 8 (47.1%) were cross-sectional, 6 (35.3%) were cohort, 2 (11.8%) were ecological, and 1 (5.9%%) was a case control. Of these 17 studies, 15 (88.2%) studies were rated as good quality, while the remaining 2 (11.8%) were rated as fair quality.

## Discussion

Building the evidence base on the impact of food environments on obesity, our review has identified for the first time a substantial number of associations between metrics of the community food environment and other health outcomes. Metrics related to unhealthy food outlets, such as fast-food outlets and convenience stores, predominantly showed positive associations with adverse health outcomes. For example, the ratio/proportion of unhealthy food outlets and presence of fast-food outlets showed predominantly positive associations with diabetes. Similarly, density of convenience stores was positively associated with diabetes and chronic disease associated mortality. Moreover, the density of fast-food outlets was consistently demonstrated positive associations with diabetes, cardiovascular diseases and chronic diseases associated mortality. This evidence is consistent with a study conducted in India [84] and Canada [85] on the association between density of fast-food outlets and overweight/obesity. This can be explained by the fact that unhealthy food outlets, such as fast-food outlets [86] and convenience stores are a key source of ultra-processed, energy-dense, and nutrient-poor foods in the community, which can contribute to suboptimal dietary behaviour [87–89]. In addition, previous studies demonstrated that, unhealthy outlets are common in low socio-economic communities [90] that typically lack healthy food options thereby encouraging consumption of unhealthy foods, with a resultant adverse effect on health outcomes [91–93]. A scoping review by Madlala et al. has demonstrated that the community food environment has a significant influence on food choice among adults in low socioeconomic communities [19].

For the first time, this review has identified that the density of full-service restaurants was negatively associated with diabetes. This is in agreement with the study conducted in Canada [85] but not with the study conducted in India [84] on the association between density of full-service restaurants and overweight/obesity. The discrepancies might be the study settings since our review is limited to high income countries. The negative association can be explained by the fact that full-service restaurant is a type of seat and dinning service which can provide a range of heathier food options [94], and is often accompanied by social entertainment and healthy food promotion [95] as highlighted in a meta-analysis [96]. In addition, full-service restaurants are typically more accessible in high socioeconomic areas, which could introduce confounding.

This review identified that living in food deserts area was predominantly associated with adverse cardiovascular outcomes. A food desert is an area characterized by low-income communities with a lack of healthy food outlets [97]. The combined effect of being unable to afford healthy foods, and lack of physical access to healthy foods contributes to unhealthy food choices [19, 92]. Beyond this, low-income areas are known to have a higher proportion of unhealthy food outlets and food insecurity which have a role in determining dietary behaviour and health outcomes [98]. Taken together, the evidence supports the hypothesis that an individual’s dietary choice is influenced by the availability, accessibility and affordability of food options whether healthy or unhealthy in their community [99]. In support of this, studies have reported that living in areas that have limited access to healthy foods including fruits, vegetables, and whole grains lead to higher rates of unhealthy eating patterns while living in the areas that have good access to fresh and healthy foods fosters healthier dietary behaviour [100, 101], and in turn, affect health outcomes [102–104].

While our review identified growing evidence of the association between metrics of the community food environment and health outcomes, a large proportion of examined associations showed null effect. This is more pronounced in metrics derived from healthy food outlets. This evidence is consistent with a previous review on the association between diet, obesity, and health outcomes [4, 17, 18, 21, 22, 105, 106]. It is likely that methodological limitations such as study designs, lower quality of included studies, diverse and unstandardized measures of the food environment, as well as varying measures of health outcomes, may be contributing factors related to nonsignificant associations. Sacks. et al. has previously identified the broad scope of food environment measures, together with diverse measures of the ways in which consumers are exposed to and interact with the food environment, along with heterogeneity of tools and methods used to measure the food environment, as key issues underpinning the association of food environment with diet, obesity, and related health outcomes [107]. The null associations may also be explained by food price differences [108, 109], and the presence of alternative means to access foods such as vehicle ownership [110, 111] and online shopping and delivery [112] which is often not well captured by community food environment studies. In addition, socio-economic status and convenience might also play an important role in determining dietary behaviour [113]. For example, a study conducted in UK showed that low food expenditure was associated with unhealthy food choices in low socio-economic groups [114]. Moreover, the mixed or null associations between the food environment metrics derived from supermarkets and grocery stores and health outcomes, might be explained by the fact that they offer a large selection of fresh and healthy food options, but that they are also sell many ultra processed, energy dense, and ready-to-use food items [11].

### Strengths and Limitations of the Review

Strengths of the review include a comprehensive search strategy, the inclusion of diverse set of food environment measures and health outcomes included in the cited studies, and rating of the quality of individual studies using design-specific standard quality assessment tools. As a limitation, while this review assessed the statistical significance and direction of association, it was unable to estimate pooled associations through meta-analysis due to numerous exposures and outcomes, as well as methodological heterogeneity. In addition, more than half of the included studies were conducted in USA, which may limit the generalizability of the findings to other high-income countries. Lastly, in order to focus on less-studied health outcomes and avoid overlap with existing reviews, obesity-only studies were excluded; however, this may have omitted some evidence about obesity as an early marker of other chronic diseases.

## Conclusion

This systematic review summarises the existing evidence on the association between community food environments and health outcomes in high-income countries. The findings underscore the growing evidence for the role of community food environment in influencing health outcomes, particularly for diabetes and cardiovascular disease. A higher density or presence of fast-food outlets and convenience stores, and a greater ratio/proportion of unhealthy food outlets often showed dominant positive associations with diabetes whereas factors such as the density of full-service restaurants, perceived availability of healthy food stores, percentage of farmers markets with direct sales, and RFEI were negatively associated. Similarly, some studies identified density of fast-food outlets and living in food desert areas were positively associated with cardiovascular diseases. Moreover, the density of fast-food outlets, density of convenience stores, and density of alcohol selling outlets demonstrated positive associations with chronic disease-associated mortality. Conversely, density of supermarkets and RFEI were negatively associated.

### Implications for Research and Practice

This growing body of evidence suggests a positive association between exposure to unhealthy food outlets and unfavourable health outcomes, and exposure to healthy food outlets being inversely associated, with implication for policy. Interventions aimed at improving the availability, accessibility, and affordability of healthy food options within communities, particularly in areas with limited access to fresh and nutritious foods, coupled with strategies towards reducing the density of fast-food outlets may be warranted.

This review highlights a need for further research to support advocacy efforts to influence local and state policies regarding urban planning and policy strategies for a healthier food environment. Future research is required that considers dimensions of the food environment using robust and consistent methodology. Standardizing and validated metrics to measures community food environment are also needed.

## Key References

- Ntarladima AM, Karssenberg D, Poelman M, Grobbee DE, Lu M, Schmitz O, et al. Associations between the fast-food environment and diabetes prevalence in the Netherlands: a cross-sectional study. Lancet Planet Heal 2022;6:e29–39.
- This study examined the association between the presence, density, and ratio of fast-food outlets within different street network buffer and diabetes, stratified by urban and rural areas.
- Corona G, Dubowitz T, Troxel WM, Ghosh-Dastidar M, Rockette-Wagner B, Gary-Webb TL. Neighborhood food environment associated with cardiometabolic health among predominately low-income, urban, black women. Ethn Dis. 31:537–46.
- This article examined the association between perceived availability, accessibility, and affordability of fruits and vegetables, shopping behaviours, and cardiometabolic health among urban low-income black women.
- Kelli HM, Hammadah M, Ahmed H, Ko YA, Topel M, Samman-Tahhan A, et al. Association between living in food deserts and cardiovascular risk. Circ Cardiovasc Qual Outcomes. 2017;10.
- This article explored the association between living in food desert areas and cardiometabolic risk factors, including diabetes, hypertension, and hyperlipidaemia.

## Supplementary Information

Below is the link to the electronic supplementary material.

Supplementary file1 (DOCX 32 KB)

## Data Availability

Data are provided in the manuscript or supplementary file.
